# Effect of oral nutritional intervention on recovery in patients with chyle leak after thyroid cancer surgery: A retrospective study

**DOI:** 10.1097/MD.0000000000046306

**Published:** 2025-11-28

**Authors:** Yongsheng Jia, Cuicui Zhang, Jimin Zhao, Zijie Niu, Xiaoyong Yang, Yan Zhang

**Affiliations:** a Department of Thyroid and Neck Oncology, National Clinical Research Center for Cancer, Key Laboratory of Cancer Prevention and Therapy, Tianjin’s Clinical Research Center for Cancer, Tianjin Medical University Cancer Institute and Hospital, Tianjin, China; b Department of Thoracic Oncology, National Clinical Research Center for Cancer, Key Laboratory of Cancer Prevention and Therapy, Tianjin’s Clinical Research Center for Cancer, Tianjin Medical University Cancer Institute and Hospital, Tianjin, China.

**Keywords:** chyle leak, low-fat diet, nutritional meal, self-controlled oral diet, thyroid cancer

## Abstract

Postoperative chyle leak after thyroid cancer surgery is a serious complication that can lead to electrolyte disturbance, malnutrition, and prolonged hospitalization. Although low-fat diet is recommended, the effects of different low-fat dietary approaches remain unclear. We compared a hospital-formulated oral nutritional meal with a self-controlled oral diet in patients with postoperative chyle leak to assess effects on recovery speed, economic burden, and electrolyte balance. This retrospective study included 87 patients diagnosed with thyroid cancer who presented with chyle leak between January and August 2022. Differences between nutritional meals and self-controlled diets were evaluated in terms of hospitalization days, postoperative expenses, drainage volume, and changes in blood sodium concentration. Patients in the nutritional meal group had significantly fewer days of postoperative hospitalization than those in the self-controlled diet group (9, 8–11 vs 11, 9–13 *P* < .05), with a significantly lower average cost of treatment (1.16, 0.99–1.45 vs 1.37, 1.16–1.81 *P *<* *.05). Maximum 24-hour drainage volume shows no significant between-group difference (232.7 ± 91.7 vs 239.0 ± 103.1 mL, *P *=* *.76), and the incidence of hyponatremia was similar (9.8% vs 6.5%, *P *=* *.12). Oral nutritional meals have clear advantages in promoting recovery and reducing hospitalization costs in patients with postoperative chyle leak after thyroid cancer surgery, suggesting that nutritional support should be strengthened in clinical practice to improve patient outcomes.

## 1. Introduction

Thyroid cancer is a common endocrine tumor, and its incidence has increased significantly in recent years.^[1,2]^ Surgery, the mainstay of treatment, typically has favorable outcomes; however, postoperative complications, especially chyle leak, remain a major clinical concern.^[3]^

The incidence of postoperative chyle leak in patients with thyroid cancer generally ranges from 1% to 8%^[4]^ and is closely related to postoperative management, surgical complexity, and underlying diseases.^[5]^ Chyle leak can lead to hydroelectrolytic disorders, infection, bleeding, and even chyle accumulation in the chest or abdomen, leading to respiratory distress and chest and abdominal infections, which can be life-threatening in severe cases.^[6,7]^ It can also prolong hospitalization and increase treatment burden. Common treatments for chyle leak include medications (e.g., growth inhibitors), localized compression bandaging, puncture drainage, and dietary interventions. Surgical repair is required for persistent chyle leak with high drainage.^[8,9]^

A low-fat diet is one of the most common conservative therapeutic measures for managing thyroid cancer and is considered to play an important role in managing chyle leak, reducing the production of chylous fluid, and improving clinical prognosis.^[4]^ Two low-fat dietary approaches are used to treat postoperative chyle leak in patients with thyroid cancer: dietitian-designed hospital-formulated oral nutritional meals and self-controlled diets.^[10]^ However, no studies have compared the therapeutic effects of these two approaches on postoperative chyle leak in patients with thyroid cancer.

Limited healthcare resources make it particularly important to assess cost-effectiveness. As healthcare expenditure increases, balancing healthcare resource allocation with patient care quality has become critical.^[11]^ During recovery from thyroid surgery, patients face complications, such as chyle leak, which not only prolongs recovery time but also triggers higher healthcare costs. Reducing the incidence of postoperative complications, which in turn reduces rehospitalization rates and healthcare costs, not only provides a better recovery experience for patients, but also reduces the financial burden on patients and healthcare systems. In this context, a cost assessment was included in this study.

We investigated the effects of different low-fat dietary approaches on the management of postoperative chyle leak following thyroid cancer surgery. Using clinicopathologic, postoperative, treatment, and cost data, we evaluated chyle leak prognosis from different perspectives to identify effective dietary strategies to improve postoperative quality of life, reduce complications, and advancing postoperative thyroid cancer treatments.

## 2. Materials and methods

### 2.1. Clinical data

Clinicopathological data of patients diagnosed with thyroid cancer who underwent surgery between January 1, 2022, and August 31, 2022, were retrospectively extracted from electronic medical records at the Cancer Hospital of Tianjin Medical University. Data collection from these records was conducted by two independent researchers between September 1, 2023, and October 31, 2023, following a predefined data extraction protocol. Discrepancies were resolved through discussion or consultation with a senior clinician. This retrospective approach enabled a comprehensive review of patient outcomes and management. The collected data included age, sex, pathological type, surgical modality, occurrence treatment of chyle leak, length of hospital stay, 24-hour maximum drainage volume, changes in postoperative electrolytes, and treatment cost. Inclusion criteria were diagnosis or confirmation of primary thyroid cancer by the hospital’s pathology department, surgical treatment for thyroid cancer, 24-hour postoperative drainage > 100 mL, and triglyceride concentration in the drainage fluid > 1.129 mmol/L. Exclusion criteria were pathological diagnosis of non-thyroidal cancer, absence of chyle leak, and chyle leak treated with adhesive injection or surgical treatment. The authors had no access to patient-identifying information during or after data collection. This study was conducted in accordance with the Declaration of Helsinki and approved by the Ethics Committee of Tianjin Medical University Cancer Institute and Hospital.

### 2.2. Treatment methods and principles

According to their specific needs, patients routinely underwent surgical treatment, postoperative wound pressure bandaging, placement of negative-pressure drainage tubes, and observation of drainage fluid changes. Patients fasted without water intake within 24 hours post-surgery. Chyle leak was diagnosed when the drainage fluid volume exceeded 100 mL and the triglyceride concentration was greater than 1.129 mmol/L. Patients with chyle leak were routinely administered octreotide (100 μg, q8 h for 2 days). Two low-fat dietary approaches were implemented: a nutritional meal and a self-controlled diet. The choice between the two approaches was made by both clinicians and patients, following a thorough explanation of the condition and the benefits and drawbacks of each one. The nutritional meal was designed by a professional dietitian, formulated by the hospital, and orally administered three times per day. The self-controlled diet comprised a low-fat diet excluding high-fat substances such as animal fats, fried foods, and milk. Patients whose drainage flow could not be controlled were treated with a high-concentration local sugar injection and surgical intervention.

### 2.3. Outcome assessment

**Figure 1.**
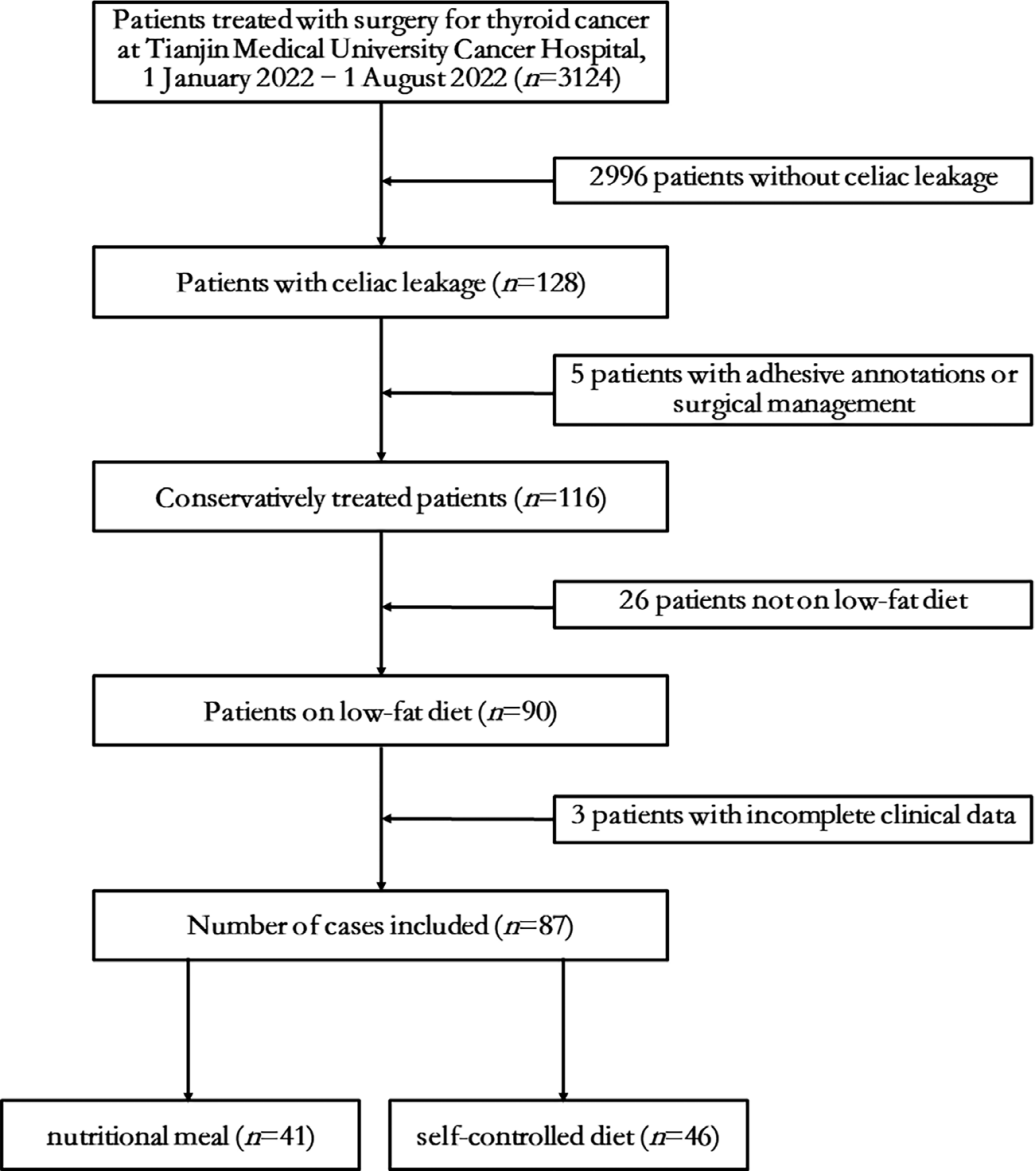
Flowchart of the screening process.

The therapeutic effects of the different dietary approaches on chyle leaks were evaluated. A total of 87 patients were successfully followed up between January 1, 2022, and August 31, 2022. The case-screening process is illustrated in Figure 1.

### 2.4. Statistical analysis

Statistical analysis was performed using SPSS software (version 23.0). Continuous variables were analyzed for normality using the Shapiro–Wilk test. Normally distributed data were expressed as mean ± standard deviation ($\bar{x}\pm s$) and compared using independent samples t-tests. Non-normally distributed variables, including postoperative hospitalization days and treatment costs, were compared using the Mann–Whitney *U* test and expressed as median (interquartile range). Categorical variables were compared using Fisher’s exact test. Count data are expressed as cases (%), with χ^2^ tests performed for intergroup comparisons. A two-sided significance level of α = 0.05 was applied.

## 3. Results

### 3.1. Clinicopathologic characteristics

Of 3124 patients who underwent thyroid cancer surgery between January 2022 and August 2022, 87 (58.6% female, median age 42 years) met the inclusion criteria (2.78%). The average hospitalization duration and treatment cost were 10.32 days (8–7) and 1.39 (0.89–3.07), respectively. A total of 41 patients received hospital-formulated nutritional meals, while 46 were prescribed a self-controlled diet postoperatively. Papillary thyroid carcinoma accounted for nearly all cases (86/87), and lateral cervical lymph node dissection was the most predominant surgical approach (86/87). Chyle leak was more frequently observed on the left side (76/87 vs 11/87). The most common type of preoperative lymph node metastasis type was lateral cervical (55/87), followed by central (31/87; Table 1).

**Table 1:** The clinicopathologic characteristics of 87 patients with post-operative chyle leak after thyroid cancer surgery.

Parameters	Number	Percentage (%)
Gender		
Male	36	41.4
Female	51	58.6
Age		
<55	66	75.9
≥55	21	24.1
Pathology		
PTC	86	98.9
FTC	1	1.1
Diet		
Nutritional meal	41	47.1
Self-controlled diet	46	52.9
Side		
Left	76	87.4
Right	11	12.6
Lymph node dissection		
Lateral	86	98.9
Central	1	1.1
Tumor size (cm)		
≤1.0	23	26.4
>1.0	64	73.6

FTC = follicular thyroid cancer, PTC = papillary thyroid cancer.

### 3.2. Comparison of the therapeutic effect of the dietary approaches

Four postoperative indicators – hospitalization days (surgery to discharge), treatment cost (postoperative period to discharge), 24-hour maximum drainage volume (highest value recorded), and blood sodium concentration changes (tested after reaching maximum drainage to monitor for hyponatremia < 135 mmol/L) – were used to assess chyle leak after thyroid cancer surgery. Reduced levels of all indicators led to quicker recovery (Table 2).

**Table 2:** A comparison of the therapeutic effects of nutritional meals and a self-administered low-fat diet on chyle leak.

	Nutritional meal	Self-directed diet	*P* value
Post-operative hospitalization days	9, 8–11	11, 9–13	.0028
Post-operative hospitalization costs	1.16, 0.99–1.45	1.37, 1.16–1.81	.0014
Drainage	232.7 ± 91.7	239.0 ± 103.1	.7648
Blood sodium level	138.2 ± 3.1	139.5 ± 3.4	.0823

#### 3.2.1. Postoperative hospitalization stay

**Figure 2.**
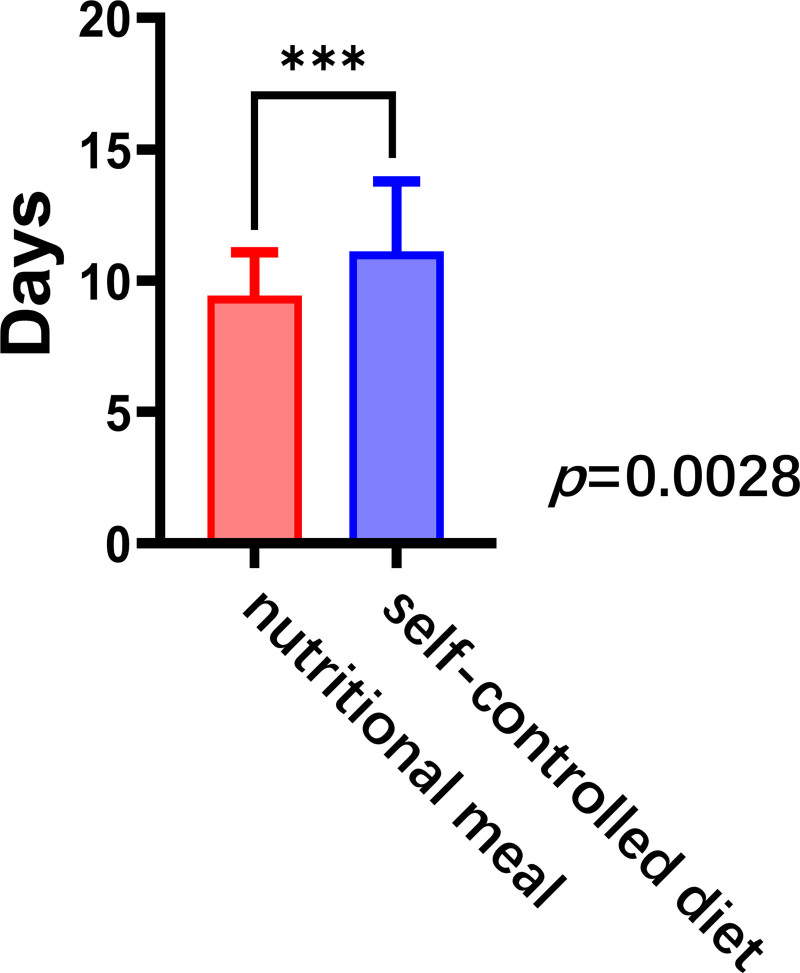
Comparison of postoperative hospitalization days between patients on different low-fat diets. ***P < .05.

Patients in the nutritional meal group had significantly shorter hospitalization days than that those in the self-controlled diet group (9, 8–11 vs 11, 9–13, *P* < .05). In comparing the rate of lateral cervical lymph node dissection between the 2 groups, no significant difference was observed between the groups (100% vs 97.6%, *P *=* *.29; Fig. 2).

#### 3.2.2. Postoperative cost

Since medical charges are not standardized, average treatment cost was used as the standard. The mean total postoperative cost for 2996 patients without chyle leak was evaluated, and the cost for patients with chyle leak was assessed by comparing their total postoperative cost with this mean.

**Figure 3.**
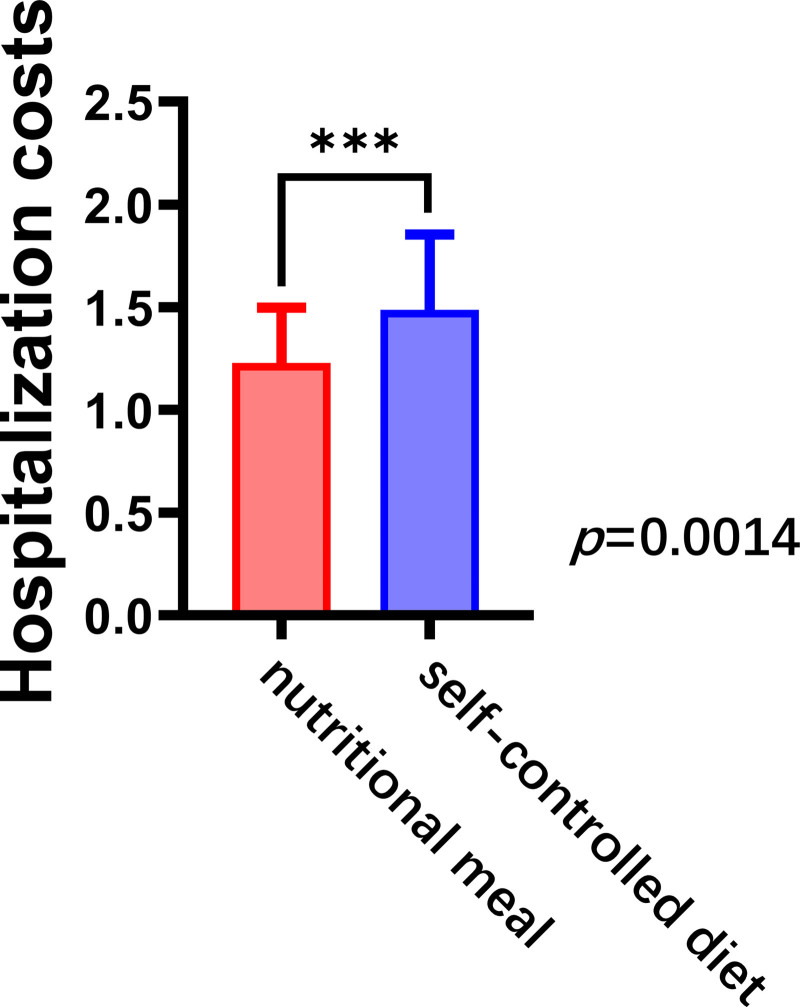
Total postoperative hospitalization costs for patients on different low-fat diets. ***P < .05.

Mean postoperative cost was significantly lower in the nutritional meal group than in the self-controlled diet group (1.16, 0.99–1.45 vs 1.37, 1.16–1.81, *P* < .05), suggesting that nutritional meals play an important role in reducing overall treatment costs; thus, nutritional meals may enhance treatment outcomes, shorten recovery cycle, and reduce financial burden (Fig. 3).

#### 3.2.3. Drainage volume

**Figure 4.**
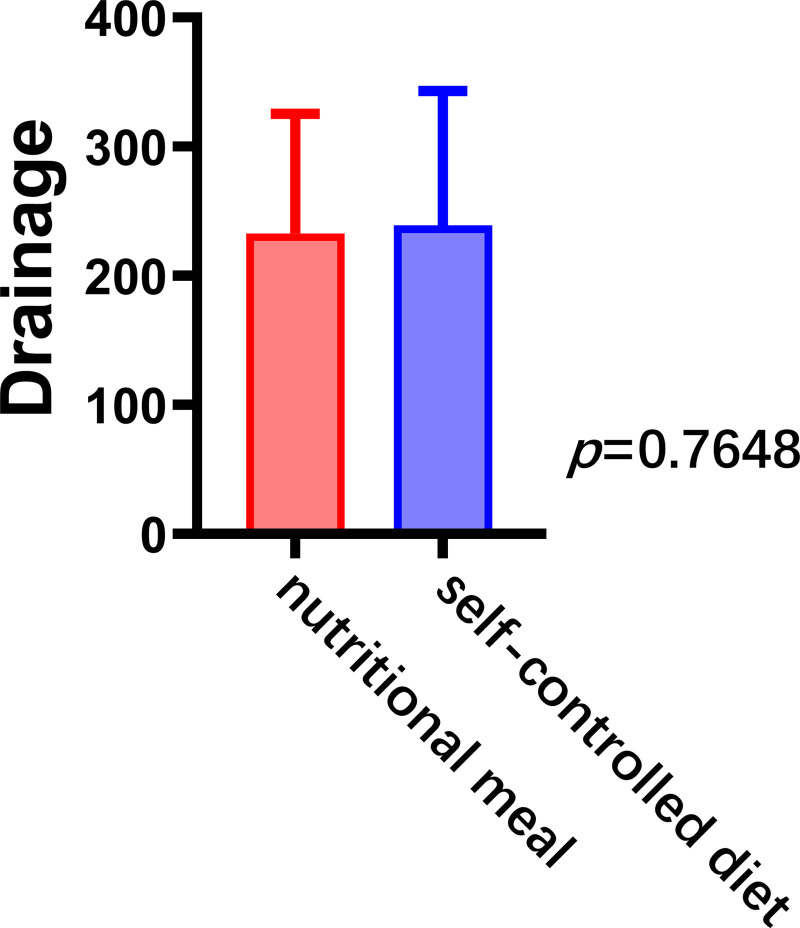
Maximum 24-hour postoperative drainage volume under different dietary regimens.

No significant difference was observed between the nutritional meal and self-controlled diet groups in terms of 24-hour maximum drainage volume (232.7 ± 91.7 vs 239.0 ± 103.1 mL, *P *=* *.76), though drainage volume was slightly in the nutritional meal group (Fig. 4).

#### 3.2.4. Blood sodium concentrations

**Figure 5.**
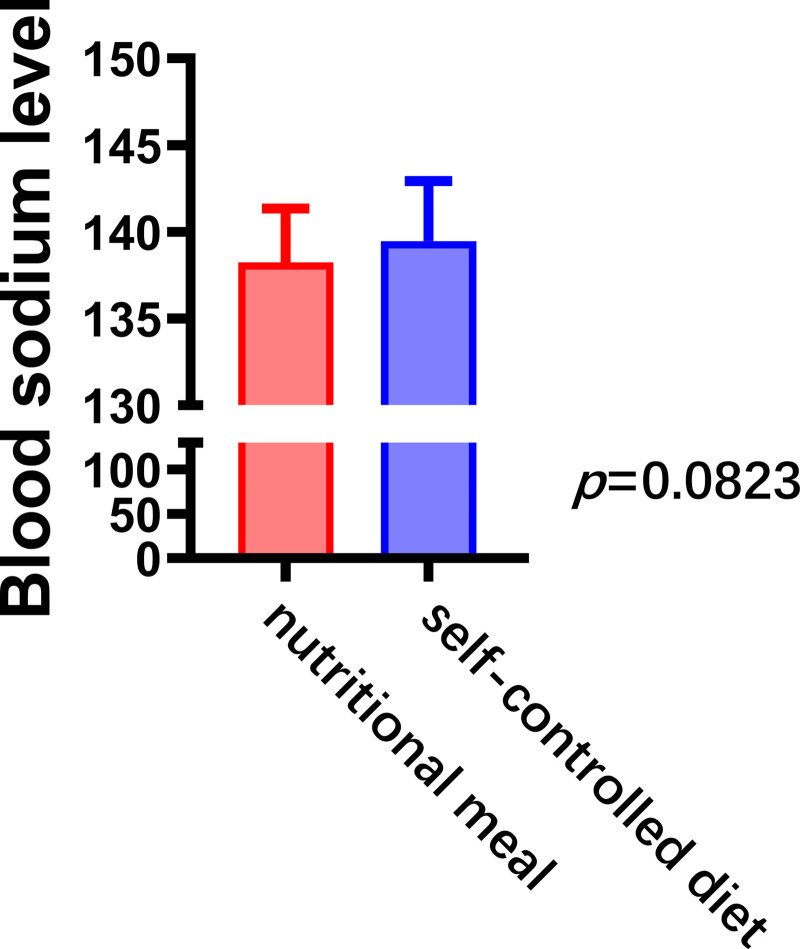
Postoperative changes in sodium concentration under different dietary regimens.

Postoperative blood sodium concentrations were higher in the nutritional meal group, with a lower proportion of hyponatremia compared with the self-controlled diet group; however, the difference was not statistically significant (138.2 ± 3.1 vs 139.5 ± 3.4, *P *=* *.08). These results suggest that nutritional meals may be more effective than self-directed dietary control in regulating electrolyte changes, though this effect was mild (Fig. 5).

## 4. Discussion

Nutritional management is an important conservative therapy for chyle leak.^[12]^ The present study focused on chylous fistula, a condition characterized by leakage of lipid-rich lymphatic fluid. Because fats, particularly long-chain triglycerides, are absorbed by the lymphatic system, a high-fat diet increases lymphatic flow and pressure. Providing a low-fat diet can reduce the burden on the lymphatic system and decrease lymphatic flow, thus reducing chyle leak and theoretically facilitating lymphatic vessel healing. By providing a complete and balanced nutritional profile, including adequate calories and protein, oral nutritional meal intervention may improve the general nutritional status of patients, which in turn might facilitate tissue regeneration and wound healing. Several approaches have been described, including total parenteral nutrition,^[13]^ medium-chain triglyceride-based enteral feed,^[14,15]^ and low-fat diets.^[4]^ Among these, an oral low-fat diet is superior to other approaches in clinical practice because it is simple and well tolerated. However, no universally established guidelines or consensus define the optimal fat content for managing this condition. Some regimens recommend a “no fat”^[16]^ or “very low-fat diet” containing less than 10 g of total fat during the initial 1 to 3 weeks, while others advance the diet to 20 to 25 g of total fat at various time points.^[17]^ In the present study, we evaluated the effects of two low-fat dietary approaches on postoperative chyle leak in patients with thyroid cancer: a dietitian-designed hospital-formulated oral nutritional meal and a self-controlled diet.

Postoperative hospitalization is a widely accepted, objective, and quantifiable measure of patient recovery. Shorter hospital stays generally reflect smoother recovery, fewer complications, and quicker readiness for discharge. It is also the most intuitive indicator of recovery from chyle leak, with fewer hospitalization days indicating faster recovery.^[4,12]^ In the present study, patients who received oral nutritional meals had significantly shorter postoperative hospitalization days compared with those who followed self-controlled diets (9, 8–11 vs 11, 9–13 *P* < .05). Further analysis revealed no significant difference in the rate of lateral cervical lymph node dissection between the 2 groups (100% vs 97.6%, *P *=* *.29). This finding suggests that nutritional meals can influence the speed of recovery in patients with chyle leakage after thyroid cancer surgery.

Beyond its socioeconomic importance, treatment cost is a determinant of financial burden on patients.^[11]^ To our knowledge, no study has been conducted to examine postoperative chyle leak treatment in patients with thyroid cancer. In this study, however, treatment cost was significantly lower in the nutritional meal group than in the self-controlled diet group, indicating a positive impact on both patients and socioeconomic burden. Implementing nutritional support may improve recovery efficiency, thus reducing future healthcare expenditures.^[18]^ From a societal perspective, lowering the cost of patient care can effectively reduce the burden on the healthcare system.^[11]^

Drainage volume is not only a diagnostic factor for chyle leak but also an indicator of recovery.^[4]^ Previous reports define drainage < 1000 mL per day as low-volume leakage, with approximately 80% of such cases improving with nutritional management alone.^[19]^ However, the roles of different nutritional support modalities in chyle leak treatment have not yet been evaluated. In the present study, no statistically significant difference was observed in 24-hour maximum drainage volume between the 2 dietary groups. The effects of different diets on postoperative chyle leak are relatively mild and, therefore, unlikely to yield rapid and significant changes in drainage volume.^[20]^ This may also be related to individual differences and the combined effects of multiple factors during postoperative recovery.^[21]^ Underlying conditions, degree of surgical trauma, and postoperative fluid balance may influence drainage volume.^[22]^

Electrolyte disorders are a common complication in chyle leak after thyroid cancer surgery, owing to the significant loss of lymphatic fluid, with hyponatremia being the most observed.^[23]^ In the present study, the incidence of hyponatremia was slightly lower in the nutritional meal group than in the self-controlled diet group (9.8% vs 6.5%, *P *=* *.61); however, the difference was not statistically significant. This phenomenon may reflect the good nutritional support received by postoperative patients.^[24]^ Based on our findings, potential clinical implications include reduced laboratory monitoring and optimization of nutritional composition.

Although this study showed differences between nutritional meals and self-controlled diets in the treatment of postoperative chyle leakage after thyroid cancer surgery, some limitations warrant further exploration and improvement. First, patients with persistent chyle leak with drainage volumes exceeding 500 mL in 24 hour rarely responded to conservative treatment and mostly underwent surgical interventions. Consequently, our analyses included only patients with chyle leak with relatively low drainage volumes, which may have affected our results. Second, postoperative low-fat diets could not be assessed because of variations in patients’ lifestyles and eating habits, potentially weakening the effect of self-directed diets. Third, the relatively small sample size may have affected the statistical reliability of the results. To improve the comprehensiveness of this study, inclusion of more patients and related indicators should be considered in future studies. In addition, the single-center design limit applicability of our results to other patient populations and healthcare settings. Multicenter collaboration, subgroup analysis, and retrospective validation should be conducted in future studies.

While our findings provide valuable preliminary evidence, further research is warranted to enhance generalizability and personalize nutritional support strategies. Specifically, future studies should prioritize the following: investigating the synergistic effects of nutritional interventions when combined with other established therapies, exploring the impact of specific fatty acid compositions, and directly comparing different modalities of nutritional support. Ultimately, future studies will pave the way for more robust evidence and sophisticated personalization of nutritional support strategies for patients with postoperative chyle leak.

## 5. Conclusions

In this study, we compared the effects of two different dietary approaches, a hospital-formulated oral nutritional meal and a self-controlled oral diet, in patients with postoperative chyle leak after thyroid cancer surgery. Our findings showed that hospital-formulated nutritional meals were associated with shorter hospitalization days and lower healthcare costs. In the current context of increasingly strained healthcare resources, effective nutritional interventions can enhance patient recovery while reducing healthcare costs. Future studies should explore the effects of different types of nutritional interventions in a diverse patient population to obtain more scientific evidence for the development of personalized nutritional support strategies.

## Author contributions

**Conceptualization:** Xiaoyong Yang, Yan Zhang.

**Data curation:** Yongsheng Jia, Cuicui Zhang, Jimin Zhao.

**Formal analysis:** Yongsheng Jia, Jimin Zhao.

**Investigation:** Jimin Zhao, Zijie Niu.

**Methodology:** Yongsheng Jia, Zijie Niu.

**Project administration:** Xiaoyong Yang.

**Resources:** Cuicui Zhang, Jimin Zhao, Yan Zhang.

**Software:** Cuicui Zhang, Jimin Zhao.

**Validation:** Xiaoyong Yang.

**Visualization:** Xiaoyong Yang, Yan Zhang.

**Writing – original draft:** Yongsheng Jia.

**Writing – review & editing:** Xiaoyong Yang, Yan Zhang.
